# The Case for Diet: A Safe and Efficacious Strategy for Secondary Stroke Prevention

**DOI:** 10.3389/fneur.2015.00001

**Published:** 2015-02-02

**Authors:** Jennifer L. Dearborn, Victor C. Urrutia, Walter N. Kernan

**Affiliations:** ^1^Department of Neurology, Yale University School of Medicine, New Haven, CT, USA; ^2^Department of Neurology, Johns Hopkins University School of Medicine, Baltimore, MD, USA; ^3^Department of Internal Medicine, Yale University School of Medicine, New Haven, CT, USA

**Keywords:** diet, secondary prevention stroke, dietary patterns, m-health

## Abstract

Diet is strongly associated with risk for first stroke. In particular, observational and experimental research suggests that a Mediterranean-type diet may reduce risk for first ischemic stroke with an effect size comparable to statin therapy. These data for first ischemic stroke suggest that diet may also be associated with risk for recurrent stroke and that diet modification might represent an effective intervention for secondary prevention. However, research on dietary pattern after stroke is limited and direct experimental evidence for a therapeutic effect in secondary prevention does not exist. The uncertain state of science in this area is reflected in recent guidelines on secondary stroke prevention from the American Heart Association, in which the Mediterranean-type diet is listed with only a class IIa recommendation (level of evidence C). To change guidelines and practice, research is needed, starting with efforts to better define current nutritional practices of stroke patients. Food frequency questionnaires and mobile applications for real-time recording of intake are available for this purpose. Dietary strategies for secondary stroke prevention are low risk, high potential, and warrant further evaluation.

## Introduction

Clinical research in nutrition for most of the past century examined the association between health and micronutrients (e.g., vitamins) or macronutrients (e.g., fat). In recent years, however, a growing body of research has also identified the importance of individual foods groups (e.g., fruit) on the incidence of cancer and cardiovascular disease including stroke (1). With substantial consistency, this work has demonstrated that higher consumption of fruits and vegetables, which are high in fiber, are associated with reduced risk for stroke (2–4).

Even more recently, investigators have turned their attention to dietary pattern and its effect on stroke risk, rather than particular food groups. Dietary patterns consumed along the Mediterranean, consisting of abundant fruits and vegetables, whole grains and low in processed foods, with a substantial polyunsaturated source of fat, such as olive oil, are consistently associated with reduced rates of stroke (5–10). The PREDIMED study, which was a multi-center randomized clinical trial comparing two Mediterranean-style diets with a low-fat diet, confirmed this association. Both Mediterranean-style diets reduced the risk of total stroke and ischemic stroke (11). These emerging data on diet and stroke risk raise the exciting possibility of a new, safe strategy for primary prevention of stroke. A great amount of work will be required, however, to confirm the promise of this strategy for secondary prevention and to develop suitable implementation methods for patients with established cerebrovascular disease. Below, we will summarize what we know about dietary patterns and stroke prevention, make some interim recommendations, and discuss how future work might help develop the potential for this therapeutic approach.

## Diet and Stroke Prevention

Numerous reviews and meta-analyses have summarized the evidence for food groups and risk of stroke, and below we highlight some important findings (2, 3, 12–14). The majority of the evidence pertains to primary prevention of stroke in large cohort studies, and focuses on micronutrients, food groups, and dietary patterns. Two themes emerge: (1) increased fruit and vegetable consumption reduces stroke risk and (2) low-fat is not better for reducing rates of stroke, and may be a harmful risk reduction strategy.

Fruits and vegetables are protective against stroke, with one recent meta-analysis demonstrating that persons with the highest fruit and vegetable consumption were 21% less likely to have a stroke compared to those with the lowest consumption [relative risk reduction (RRR), 0.79; 95% confidence interval (CI), 0.71–0.84] (2). In the meta-analysis, the association was similar for fruit and vegetable consumption when considered separately. This is comparable to the effect size for statin use for prevention of stroke in high-risk patients, where statin use reduced all strokes by 18% (95% CI, 13–23%; *p* < 0.0001) (15). Nine large prospective cohorts with unique populations, such as the Nurses’ Health Study, the Danish Diet, Cancer and Health Study, and the Framingham cohort among others add to the compelling evidence (16–20) that increasing fruit and vegetable consumption is associated with reduced burden of stroke. The INTERSTROKE study, designed to describe risk factors associations with the global burden of stroke, also demonstrated that fruit was associated with a lower risk of ischemic stroke (4). Across diverse populations with unique dietary patterns, mounting evidence is indicating that higher consumption of plant-based food-items is associated with reduced risk for stroke.

The evidence is not as consistent for other food groups (such as meat, dairy, and eggs) and incident stroke, but fish consumption seems to be beneficial. Overall, evidence suggests a modest protective effect of fish consumption and incident stroke (4, 21–23), although some studies found no associations (24). This may depend on the way fish is eaten, as in many cultures fish is salted, which may negate its beneficial effects. One meta-analysis of 15 prospective studies reported a 6% reduction in stroke in by consuming an increment of three or more servings of fish per week (RRR, 0.94; 95% CI, 0.89–0.99) (21). Japan, which has a much lower consumption of animal products and higher fish consumption than Western countries, has seen increasing consumption of meat, dairy, and eggs since the 1960s and during this time an associated reduction in cerebrovascular mortality. It is unclear if the increased consumption of animal products is truly protective, or if other dietary changes, such as a reduction in sodium, could account for this difference (25–27). In other cohorts, total meat consumption may be associated with a higher risk of ischemic stroke (12), however as meat consumption increases, it is likely that consumption of other beneficial food groups (such as fruits and vegetables) decreases.

There is increasing evidence that processed foods and meats may contribute to cardiovascular risk (28). Preservation of processed meats, such as ham, cold cuts, and bacon, occurs by smoking, curing, salting, or the addition of chemical preservatives. Although all studies do not share positive associations with stroke incidence and consumption of processed meats, a meta-analysis suggests an association exists (28, 29). The link between dairy foods, such as milk, cheese, and butter, and stroke is unclear, as no associations have been reproducibly demonstrated (30).

The association between dietary fat and risk for stroke is complex. Dietary fat is discussed in terms of: total fat, saturated, monounsaturated or polyunsaturated fat, or trans-fat. Saturated fats are found primarily in animal products, such as meat and dairy. Mono or polyunsaturated fats are found in vegetable oils and fish. Trans-fats are synthetic and found in margarine and other processed foods. Total fat, or other types of fat, were not associated with incident stroke in a 14-year follow-up study of US male healthcare professionals (31). Consistent with this observation, a randomized trial within the Women’s Health Initiative demonstrated that a dietary modification to lower total fat also did not reduce stroke risk (32).

If a low-fat diet is not optimal for reduction of stroke risk, the type of fat may be important (22, 33–35). The U.S. Department of Agriculture released the food pyramid in 1992 to emphasize that “fat is bad,” largely ignoring epidemiologic data about different types of fat. Nutrition science has moved beyond the pyramid, but its iconic message continues to influence public perceptions of “healthy eating” (36). One large meta-analysis suggested that saturated fat, previously recommended for lower intake to reduce cardiovascular disease incidence, was not associated with cardiovascular disease or stroke (37). These controversial results (38) have been supported by a large cohort, which showed a reduction in stroke risk with increased consumption of saturated fat in men (39). Evidence also suggests that increased consumption of certain types of polyunsaturated fats, such as long-chain omega-3 polyunsaturated fatty acids, may lower stroke risk (35) Trans-fats, on the other hand, may be associated with increased rates of stroke, at least in men (34). The effect of fat replacement (i.e., replacing saturated fats in the diet with polyunsaturated fats) has not been evaluated in stroke. The new initiative by the US Department of Agriculture, My Plate, has replaced the pyramid and shifts emphasis from lowering fat consumption to increasing relative consumption of fruits, vegetables, and whole grains (40). With existing data, it is difficult to make firm recommendations about fat consumption in stroke patients, and the best data will likely come from studies that incorporate whole dietary patterns (33).

Dietary patterns look at foods groups consumed together, rather that examining associations between individual foods and outcomes (41). Five large observational studies suggest that the Mediterranean-style diet: rich in fruits vegetables, low in red meat, with moderate alcohol, and use of olive oil or non-hydrogenated fats, may have the most potential as a dietary intervention to prevent stroke (5–11, 42). Prospective cohorts including the Nurses’ Health Study, Northern Manhattan Study, and the European Investigation into Cancer, all demonstrate reduced stroke risk with this pattern (7, 9, 10). A secondary prevention study in Lyon, France, also supported the potential of the Mediterranean diet to reduce recurrent cardiovascular events, however stroke was included as a part of a composite outcome (43). The dietary approaches to stop hypertension (DASH) diet, which similar to the Mediterranean diet, emphasized fruits, vegetables, as well as low intake of red meat and sweets, has also shown a benefit for stroke risk reduction (44). Another dietary pattern described in cohort studies is the Prudent diet, which also has a higher intake of fruits and vegetables, whole grains, legumes, and fish. Similarly, this dietary pattern has been associated with reduced risk of stroke (45).

There is almost no data on what stroke patients eat, either before or after their stroke. The majority of studies in diet and nutrition post-stroke have focused on measures of malnutrition or under-nutrition (46–49), rather than diet quality, diet composition, or eating habits. In the absence of this information, it is difficult to identify potential opportunities for improvement. We do know that stroke may result in neurological complications (e.g., dysphagia, loss of motor control, and depression) that may increase risk for malnutrition. Aphasic patients may not be able to communicate when they are hungry or what they want to eat, and prolonged hospitalization may alter metabolic function and metabolic needs. Designing a nutritional intervention must take account of these complications for individual patients.

## Importance of Diet for Regional Differences in Stroke Risk

Variation in dietary pattern between geographic regions may be one explanation for geographic variation in stroke risk. The “Stroke Belt” in southeastern United States defines an 11-state region with particularly high rates of stroke and cardiovascular disease, including an area ranging from North Carolina to Louisiana (50, 51). The NINDS-funded REGARDS study is an observational study designed to understand why people in this part of the country are at a higher risk of stroke. A dietary pattern, known as the “Southern” pattern has been described in the REGARDS study; it is high in fried foods, organ and processed meat, and sugar-sweetened beverages (52). This dietary pattern is associated with increased incidence of stroke in this region. The southern diet is an example of diet preferences (such as sweet tea) that may be similar within neighborhoods because of ethnic backgrounds, economic reasons, or be due to food choice and accessibility of restaurants and grocery stores (53–55). It is of no surprise that, along with diet, obesity, and chronic diseases including diabetes and cardiovascular disease cluster within neighborhoods, and are especially prevalent in minority communities (56–58). Increased rates of stroke are described in impoverished neighborhoods as compared with more affluent communities (59–62). Dietary patterns may be part of the causal pathway in “high-risk” communities with high rates of stroke (5, 6, 52, 63).

There is significant geographic variability in type of fats consumed. Asian countries such as China, Japan, and South Korea have the lowest per-capita consumption of saturated fats, while in many Latin American countries, consumption of more beneficial polyunsaturated fats may be low (64). Some Pacific island nations have high intakes of palm oil, which is known to be high in saturated fat, and therefore have low polyunsaturated fat consumption. Higher consumption of seafood, rich in omega-3 polyunsaturated fat, is present in the Mediterranean, Iceland, South Korea, Pacific Islands, and Japan, however is extremely low in many other parts of the world, including Sub-Saharan Africa and South America (65). The contribution of these differences in fat consumption to incident stroke is largely unstudied.

Improved population data is facilitating research on the association between diet and stroke risk in low, middle, and high-income countries (66). One must be cautious in interpreting this research, however, because of potential confounding by factors such as air pollution, smoking rates, and genetic risk. Greece, for example has higher stroke mortality than Sweden despite a diet that should afford more protection against vascular disease (66). With careful characterization of diet at a regional level, and rigorous examination of possible confounders, more accurate conclusions about the aspects of diet that explain regional differences can be understood. Given the lack of detailed dietary information in many regions (for example urban versus rural US populations), there is a need for an accurate, convenient method for quantifying diet for populations at risk of stroke. Technology, specifically mobile health applications, may play a role in defining the phenotype.

## Methods for Measuring Dietary Pattern

There are three common methods to record a person’s diet: a dietary record, 24-h diet recall and food frequency questionnaires (FFQs) (67). Dietary records document foods as they are consumed, usually for 3 or 4 days. They may be affected by error if respondents modify or systematically underreport food eaten, or if compliance is non-random. The 24-h diet recall is completed as a patient writes down foods that they have consumed in the past 24 h. This method is simple to administer, and does not require an intensive time commitment. It may not be accurate, however, if the sampling interval is not representative of the respondent’s typical diet.

Food frequency questionnaires are commonly used in large cohort studies and ask a participant to report how often he or she consumes food items over an average time frame, such as the past week (68). Several FFQs have been validated for use. These tools are simple to administer and semi-quantitative in that total daily intakes of food groups can be estimated (69–71). A typical FFQ includes 60–200 items, including simple foods, like apples or carrots, or complex foods specific to the region studies, such as grits or lasagna. However, this, too, is a rough estimate of an individual’s diet pattern, and may be affected by personality characteristics that lead to over or under reporting. This is often the method of choice in cohort studies because of ease of administration, and reproducibility, but its validity is determining actual consumption is limited (72). Measurement error in the tool of choice, can lead to missed associations with chronic disease (73).

Food frequency questionnaire and 24-h recalls are commonly used to classify specific eating patterns using *a priori* diet scores. One example is the Mediterranean Diet Score, based on foods typically eaten in the Mediterranean region, which creates a numeric score of 0–9, based on nine food group items, where a subject would get a point for each food group if they are above the median value of the sex-specific consumption for beneficial food-items (74). Diet scores have also been created for measuring adherence to US Government dietary recommendations (33), consumption of carbohydrates (75, 76), and adherence to the DASH diet (44, 77) (Table 1). The benefit of a diet score is that it may allow a hypothesis driven approach to study outcomes. For example, the Healthy Eating Index was created to study the nutrient needs and dietary guidelines of the US consumer, with the potential for the US Department of Agriculture to incorporate it into health promotion methods (78). This index incorporates a 10-component score of 5 food groups, 4 nutrients, and 1 measure of the variety of food intake. The DASH diet score allows a dietary intervention developed as a part of a randomized controlled trial (79) to be used in a prospective community-based cohort, by incorporating data from a FFQ.

**Table 1:** **Summary of stroke studies including *a priori* and principal components analysis for defined dietary patterns**.

Cohort	*N*	Dietary patterns	Associations with stroke
Male health professionals, Nurses (33)	38,615	Alternate healthy eating index (AHEI)	Stroke grouped with all cardiovascular disease (CVD)
	67,271	Recommended food score (RFS)	High AHEI score associated with reduced risk of CVD
			RFS predicted risk of CVD in men but not women
Europeans (6)	47,021	Healthy eating index (HEI); dietary approaches to stop hypertension (DASH); Greek Mediterranean index; Italian Mediterranean index	Three indices (not HEI) associated with lower risk of stroke, with the greatest risk reduction for the Italian Mediterranean index
Europeans (75)	44,099	Glycemic index and glycemic load	High glycemic index associated with increasing risk of stroke
Europeans (9)	40,011	Mediterranean diet	Better adherence to Mediterranean diet inversely associated with stroke
New York city residents (10)	3,298	Mediterranean diet	No association between Mediterranean diet and risk of ischemic stroke
Nurses (7)	74,866	Mediterranean diet	Greater adherence to the Mediterranean diet associated with a lower risk of stroke
Nurses (44)	88,517	DASH	DASH score associated with lower risk of stroke
Nurses (76)	78,779	Glycemic index and glycemic load	Carbohydrate intake associated with increased risk of hemorrhagic but not ischemic stroke
Nurses (45)	121,700	Prudent and Western	Western pattern associated with higher risk of stroke; prudent pattern trended toward a lower risk of stroke
U.S., oversampled Southeast residents (52)	30,239	Convenience; Southern; plant-based; sweets/fats; alcohol/salads	Southern style diet may increase stroke risk; plant-based diet may reduce stroke risk
Multi-ethnic U.S. population (80)	6,814	Fat and processed meat; vegetables and fish; beans, tomatoes, and refined grains; whole grains and fruit	Stroke grouped with CVD; fat and processed meat associated with higher risk of CVD; whole grains and fruit associated with lower hazard CVD

Unlike *a priori* diet scores, which develop from hypotheses about food groupings, principal components analysis is a data-driven approach used to examine food consumed in individuals that are correlated. The goal is to break the food groups into a smaller amount of groupings of correlated components, called clusters, to describe the diet patterns with fewer variables (81). As each principal components analysis is specific to the cohort studied, the generalizability to other populations can be questioned. However, within each cohort, they can elucidate patterns of eating associated with disease outcomes that incorporate the total diet. Examples of dietary patterns described are a “Prudent” pattern (high in fruits and vegetables, “low in red meat”) and a “Western” pattern, which is high in red meat, processed foods, and sweets (45). It is important to compare this approach to analyses across cohorts to determine if the associations of different patterns with stroke have similar food groupings. Table 1 shows the major studies that have examined dietary patterns with ischemic stroke as the outcome (6, 7, 9, 10, 39, 44, 45, 52, 76, 80).

Portion size is defined by the amount of food a person chooses to eat at a given time. Recommendations by groups such as the U.S. Department of Agriculture are based on serving size, which is a standard amount of food recommended for consumption. In epidemiologic and clinical studies, it is challenging to measure portion size because of systemic bias in over or under reporting. This leads to errors in total energy calculations, which are shown to be underreported in nationwide surveys (82). Therefore systematic research on portion size and disease outcomes, such as stroke is lacking. We do know however, that for weight loss, which is associated with reduced incidence of cardiovascular disease, the total amount of calories seems to be more important than the composition of the macronutrients (i.e., proportion of carbohydrates, fats, and protein) (83).

## Emerging Mobile Technologies

Mobile technology provides an opportunity to better measure dietary consumption because of several key factors: (1) it is widely available and commonly used for other purposes (namely mobile phones), (2) in modern society most always have a mobile phone at hand, which is an opportunity to record data at the moment of food consumption, and (3) mobile phones have the ability to wirelessly transmit data collected (84). These features are excellent for stroke research because they can improve the accuracy of dietary data collected and ensure that it is collected in a standardized format ready for analysis. The ubiquitous mobile phone would allow individuals in harder to reach or understudied/minority communities to participate in research so that the full phenotype of dietary patterns can be discovered.

The mobile health (m-health) revolution began in a response to these observations, and m-health strategies have been employed in a variety of applications in the developing world and in harder to reach communities (85). One of the successes in m-health applications has been in diabetes education, in which patients are able to record food records and blood sugars, which can be transmitted to researchers and doctors (86). Many of the interventions invoke cell phone text messages, known as Short Message Service (SMS), to remind patients about blood sugar control, and show moderate efficacy in supporting patient’s self-management (87). Other mobile applications seek to detect atrial fibrillation in asymptomatic patients in the community by a single lead EKG embedded in an iPhone (88). Dietary interventions are currently proposed using m-health to improve fruit and vegetable consumption in young adults (89). Other than primary prevention of cardiovascular diseases or diabetes, data on m-health applications in stroke is growing in areas such as telemedicine and before hospital triage, but applications in diet and secondary prevention are lacking (90).

**Figure 1:**
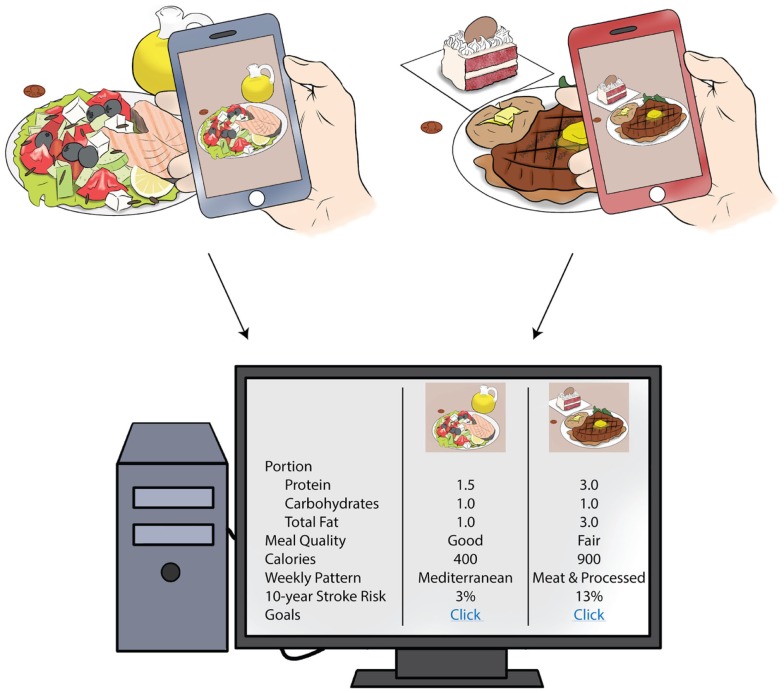
**Mobile health, diet, and potential clinical applications**. Above is an example of how patients can use mobile health technology to record dietary intake to be interpreted in the clinic setting. Using smart technology, daily intake and portion size could be calculated for foods consumed. The computer algorithm could determine the overall meal quality, based on dietary recommendations, and the dietary pattern that the person adheres to. Patients could be counseled on his or her stroke risk attributable to diet, based on information from population-based studies. This information could lead to individualized diet recommendations for stroke prevention.

An exciting application that could have particular relevance to diet and stroke prevention involves mobile nutrition records that are recorded via photographs of foods being eaten (91, 92). Each photograph is set with a fiducial marker so that measurement of portion size can be accurate, and the photograph is compared with a database of foods for similarities so that portion size and foods can be identified. User interaction and confirmation of foods and portion sizes is still critical, but can often be done after the meal if the image is captured. This technology is still investigational and requires validation with other food recording methods, but is very promising because it allows for standardization and automatization of food portion sizes and calorie counts between subjects. This no longer relies on individuals to “estimate” portion sizes and total calories. It has the potential to be more “user-friendly” by allowing participants to take a picture, rather than search for foods consumed through cumbersome electronic food records (93, 94). Figure 1 shows a potential application of this technology in a patient population. It is clear that more rigorous validation of mobile health food records is needed in comparison with the FFQ and other techniques, but the benefits to the user of such methods are evident.

## Future Directions and Conclusion

Statin use has been widely incorporated into clinical recommendations and quality control measures in the care of patients with stroke, based on available evidence that demonstrates efficacy in secondary prevention. A healthy diet may have a similar effect size for secondary prevention but is hardly mentioned during hospital admissions and clinic follow-up visits for stroke patients. This may be because there is no direct research data to show that any specific diet is more effective than another in secondary stroke prevention. There is also a lack of support for nutritional counseling in many clinics and hospitals. Finally, even with adequate knowledge and support, patients have great difficulty adopting new dietary patterns. These three challenged are not insurmountable, but overcoming them will require research on nutritional epidemiology in stroke, comparative effectiveness of various diets, and implementation.

As a reasonable first step, better characterization of diet phenotype in stroke patients may reveal specific opportunities for improvement and suggest further research directions. If most stroke patients eat poorly, for example, then efforts to intervene might gain attention. If the research shows common adverse dietary practices in stroke patients within the same geographic region, then community-based interventions might gain attention. Only carefully designed survey research, furthermore, will identify barriers to optimal nutrition in stroke patients and provide the information that is needed to design and test effective implementation strategies.

## Conflict of Interest Statement

The Review Editor Shaida A. Andrabi declares that, despite being affiliated to the same institution as author Victor C. Urrutia, the review process was handled objectively and no conflict of interest exists. The authors declare that the research was conducted in the absence of any commercial or financial relationships that could be construed as a potential conflict of interest.
